# Scientific Items

**Published:** 1888-02

**Authors:** 


### SCIENTIFIC ITEMS.

**Artificial Silk.**—The author dissolves 3 grms. nitro-cellulose in 100 to 150 c. c. of a mixture of equal parts of alcohol and ether. He adds 2-5 c. c. of a filtered solution at one-tenth of the dry ferrous chloride of commerce in alcohol, or of stannous chloride, and further 1·5 c. c. of a solution of tannic acid in alcohol. The whole is filtered in a closed apparatus to prevent loss by evaporation. The liquid is placed in a vertical reservoir, having at its bottom a blowpipe nozzle of glass or platinum. This pipe forms an acute cone with an orifice of from 0·10 to 0·20 mm., the thickness of the margin not exceeding 0·1 mm. This aperture opens into a vessel of water acidulated with one-half per cent. of mono-hydrated nitric acid. The level in the reservoir being some centimeters higher than in the vessel of water, the outflow proceeds easily. The fluid thread hardens at once in the acidulated water, and may be drawn out by a uniform movement. The thread thus formed must be dried rapidly by traversing a current of dry (not hot) air, and may be wound up soon as dry. It is gray or black, but a number of soluble coloring matters may be introduced into the etheral solution, thus obtaining threads of all colors.—*M. de Chardonnet.*

**A New Gas.**—The discovery of a new gas is reported in Germany by Dr. Theodore Curtins, who has succeeded in preparing the long sought hydride of nitrogen, amidogen, diamide, or hydrazine, as it is variously called. This remarkable body, which has hitherto baffled all attempts at isolation, is now shown to be a gas perfectly stable up to a very high temperature, of a peculiar odor (differing from that of ammonia), exceedingly soluble in water, and of basic properties. In composition it is nearly identical with ammonia, both being compounds of nitrogen and hydrogen.

**Do Horses Have Horse Sense?**—A writer in the *Chicago Journal* thinks the intelligence of the horse is greatly over-rated, and submits the result of some of his observations as evidence:

"I have seen horses walk around a post until they had wound up the bridle, and then stand all day with their heads down to the post, because they didn't have sense enough to walk the other way, and unwind the bridle. I have seen them prance around in a burning barn, with the tails and manes on fire, and burn to death, because they did not have sense enough to run out. Anybody can steal a horse without any objection from the horse. A horse will stand and starve and freeze to death, with nothing between him and a comfortable stall and plenty of oats, except an old door that he could kick down with one foot, or that could be opened by removing a pin with his teeth. If this is a high degree of intelligence, even in a brute, then I am lacking in that article myself. Compared with the dog, the elephant, or even the parrot, the horse seems to me to be a perfect fool.—*Popular Science News.*

**Changes in Teeth.**—Dr. B. C. Windle, of Dublin, in a paper read before the British Dental Association on "Man's Lost Incisors," reaches the following conclusions:

1. Man's original dentition included six incisors.
2. Man's lost incisors are the lateral.
3. This loss is consequent upon the contraction of the anterior part of the jaw.
4. Suppression of the two present lateral incisors is now taking place.
5. Conical teeth are a reversion to the primitive type.—*Ib.*

**The** recent discovery of the power possessed by soaring birds to set their wings when fully expanded, and to remain locked independent of muscular action, explains to my mind a phenomenon that has puzzled me for many years. It has been my custom for many seasons to spend a few days each fall duck-shooting at the lakes bordering the Illinois River in central Illinois. The birds were almost invariably shot in mid-air, while flying rapidly by, and often, when not killed at once, they would set their wings and sail gradually down to the water or ground, which they would reach dead, the distance being from one hundred yards to a quarter of a mile, apparently corresponding to the height of the bird when shot. And it was a maxim with duck-shooters on these lakes, "That bird is killed, for he has set his wings."

Besides the ducks, I have seen this phenomenon illustrated in the wild turkey and prairie-hen. In wing-shooting the wild turkey, if it set its wings, and gradually came to the earth a quarter of a mile or more away, we always marked the spot, well expecting to find the dead body when we reached it.—*Science.*

**The following illustration** of the practical usefulness of bacteriology is given by the *New-York Medical Record:*

"An Italian steamer arrived loaded with immigrants. There had been no cholera on board; but as the vessel reached this port, a suspicious case of diarrhœa occurred in a child. The symptoms were not perfectly typical of cholera. Some of the dejections were taken, and sterilized tubes were inoculated, and taken to the Carnegie Laboratory in this city. It would take four days to develop the cultures; and the question arose, whether the steamer should be delayed for that period of time. It was finally decided to do so. The cultures developed in the way characteristic of Asiatic cholera, and the diagnosis was made. Subsequently other cases of cholera appeared, and the culture-diagnosis was abundantly confirmed.—*Ib.*

**Mr. Edison** now claims that he has so improved his phonograph as to make it a practical machine for correspondence, and prophesies that it wil entirely supplant the typewriter and similar methods for facilitating business. Instead of writing or printing a communication on a sheet of paper, the words will be spoken before the diaphragm of the phonograph, and the vibrations registered on a small piece of tinfoil, which can be sent by mail, and when placed in another phonograph, will reproduce the words as originally spoken.—*Ib.*

